# Proteomic analysis of media from lung cancer cells reveals role of 14-3-3 proteins in cachexia

**DOI:** 10.3389/fphys.2015.00136

**Published:** 2015-04-28

**Authors:** Julie B. McLean, Jennifer S. Moylan, Erin M. W. Horrell, Francisco H. Andrade

**Affiliations:** ^1^Department of Physiology, University of KentuckyLexington, KY, USA; ^2^Center for Muscle Biology, University of KentuckyLexington, KY, USA; ^3^Center for Clinical and Translational Science, University of KentuckyLexington, KY, USA; ^4^Markey Cancer Center, University of KentuckyLexington, KY, USA

**Keywords:** cachexia, cancer, myosin, proteomics, skeletal muscle, 14-3-3 proteins

## Abstract

**Aims:** At the time of diagnosis, 60% of lung cancer patients present with cachexia, a severe wasting syndrome that increases morbidity and mortality. Tumors secrete multiple factors that contribute to cachectic muscle wasting, and not all of these factors have been identified. We used Orbitrap electrospray ionization mass spectrometry to identify novel cachexia-inducing candidates in media conditioned with Lewis lung carcinoma cells (LCM). Results: One-hundred and 58 proteins were confirmed in three biological replicates. Thirty-three were identified as secreted proteins, including 14-3-3 proteins, which are highly conserved adaptor proteins known to have over 200 binding partners. We confirmed the presence of extracellular 14-3-3 proteins in LCM via western blot and discovered that LCM contained less 14-3-3 content than media conditioned with C2C12 myotubes. Using a neutralizing antibody, we depleted extracellular 14-3-3 proteins in myotube culture medium, which resulted in diminished myosin content. We identified the proposed receptor for 14-3-3 proteins, CD13, in differentiated C2C12 myotubes and found that inhibiting CD13 via Bestatin also resulted in diminished myosin content. Conclusions: Our novel findings show that extracellular 14-3-3 proteins may act as previously unidentified myokines and may signal via CD13 to help maintain muscle mass.

## Introduction

Lung cancer kills more people each year than prostate, pancreatic, breast, and colon cancers combined (Society, 2012). At the time of diagnosis, 60% of lung cancer patients have cachexia, a severe wasting syndrome that includes loss of muscle mass, weakness, and fatigue (Fox and Wang, 2007; Fearon et al., 2011; Society, 2012). This syndrome cannot be reversed by nutritional interventions, diminishes response to and tolerance of cancer treatments, and increases morbidity and mortality (Fearon et al., 2013). Over the last several decades, research has revealed the complex and multifactorial nature of cachexia (Tan and Fearon, 2008; Blum et al., 2011; Fearon et al., 2011; Tsoli and Robertson, 2013). Tumor- and host-derived inflammatory factors stimulate degradation of myofibrillar proteins via signaling pathways, including up-regulation of ubiquitin proteasome activity and autophagy (Romanello and Sandri, 2010; Fearon et al., 2013). In addition to increased protein degradation, protein synthesis is also reduced, contributing to a net loss of muscle mass (Gordon et al., 2013). The intracellular pathways leading to muscle wasting have been widely studied; however, we know less about the tumor-derived extracellular factors that alter the balance between protein synthesis and degradation. Although cachectic cytokines, such as TNF-α and IL-6, are elevated in the sera of cancer patients, clinical trials with one-target inhibitors have proven ineffective (Mantovani et al., 2013; Tsoli and Robertson, 2013). These results confirm that no single cytokine is responsible for cachexia, and suggest that there may be unknown extracellular factors contributing to cancer-induced muscle wasting. Identifying novel cachectic factors warrants further study.

We chose to focus on identifying cachectic factors from lung cancer because it accounts for 23% of all cancer deaths worldwide (Jemal et al., 2011). Lewis lung carcinoma served as our cancer model because it is known to induce cachexia both *in vitro* and *in vivo* (Carbo et al., 2004; Argiles et al., 2008; Puppa et al., 2014). One hundred and fifty eight proteins were identified by mass spectrometry, and we focused on the 33 secreted proteins. 14-3-3 proteins in particular captured our attention: they are a multi-functional and highly conserved family of binding proteins with over 200 known partners (Freeman and Morrison, 2011; Gardino and Yaffe, 2011; Kleppe et al., 2011; Obsil, 2011). The seven isoforms of 14-3-3 proteins are routinely found in the intracellular environment affecting signaling pathways by altering enzymatic activity, protein-to-protein interactions, cellular location, and protein stability (Freeman and Morrison, 2011; Gardino and Yaffe, 2011; Kleppe et al., 2011; Obsil, 2011; Tzivion et al., 2011). Several recent studies have shown that some isoforms, including 14-3-3η, 14-3-3σ, and 14-3-3α/β, act in an extracellular manner to activate signaling cascades (Ghaffari et al., 2010; Asdaghi et al., 2012; Maksymowych et al., 2014). We found that depletion of extracellular 14-3-3 proteins decreased myosin content in skeletal muscle. Extracellular 14-3-3 proteins potentially represent a novel mechanism of regulating skeletal muscle mass.

## Materials and methods

### Myotubes

We plated C2C12 myoblasts (American Type Culture Collection) at a density of 10,000 cells/cm^2^ in growth medium [Dulbecco's modified Eagle's medium (DMEM) with 10% fetal bovine serum (FBS), 1.6 g/L NaHCO_3_, and 100 U/ml PenStrep (Invitrogen)] and grew cells at 37°C in 5% CO_2_. After 3 days, cells reached ~90% confluence, and we serum-restricted the cells in differentiation media (DMEM as above with 2% horse serum replacing FBS). After 4 days in differentiation media, multinucleated myotubes were ready for treatment. Fresh medium was added every 2 days (Moylan et al., 2014).

### Cancer cells

Lewis lung carcinoma cells (LL/2: American Type Culture Collection) were seeded in 100 mm cell culture plates in growth medium (as above) at a density of 6000 cells/cm^2^. After 2 days, we added supplementary growth media to each plate. LL/2 cells contain a heterogeneous mix of adherent and floating cells. After 4 days, we removed growth medium, and floating cells were harvested by centrifugation at 500 × g, 5 min. Pelleted cells and 10 mL differentiation media were added back to the plate containing the adherent cells. After 2 days, conditioned media were harvested and cleared of cells and debris by centrifugation (500 × g, 5 min). Aliquots were frozen in liquid nitrogen for later use. For myotube treatments, conditioned media were diluted 1 in 4 with fresh differentiation media. For mass spectrometry analysis, serum-free media replaced differentiation media (McLean et al., 2014).

### Western blot

We homogenized C2C12 myotubes in 2X protein loading buffer (120 mM Tris pH 7.5, 4% SDS, 200 mM DTT, 20% glycerol, 0.002% bromphenol blue). Proteins were separated in equal volumes of lysates by SDS-PAGE (4-15% Criterion, BioRad). We determined relative total protein by scanning (Odyssey Infrared Imaging, LI-COR) stained gels (Simply Blue, Invitrogen). We used fluorescence intensity data to normalize total protein for equal loading. SDS-PAGE was used to separate equal amounts of protein, which was then transferred to PVDF membranes for western blot using the Odyssey System (Moylan et al., 2014). For conditioned media samples, we combined 6X loading buffer (as above) 1:10 with conditioned media, and followed the same procedure as for lysates.

### Antibodies

For western blot, primary antibodies were mouse anti-myosin (Sigma), rabbit anti-pan 14-3-3 (Cell Signaling), and rabbit anti-CD13 (Abcam). Secondary antibodies included anti-mouse IRDye 800 CW and anti-rabbit IRDye 800 CW (LI-COR). For antibody neutralization experiments, we treated cell culture medium with a 1:200 dilution of the primary antibody for pan-14-3-3 or CD13, or the respective heat-inactivated control for 48 h.

### Fluorescence microscopy and width measurements

For width measurements, we incubated C2C12 myotubes with nucleic acid stain, Syto13 (2.5 μM, Invitrogen) for 15 min. We captured live-cell bright field and fluorescence images (excitation: 488 nm, emission 509 nm) using a CCD camera (CoolSNAP-ES, Roper Scientific Photometrics) attached to a Nikon TE2000 microscope with NIS Elements image acquisition software (Nikon). To obtain the average myotube width per well, we measured 3 images per well, 5 myotubes per image, and 5 width measurements per myotube. For CD13 immunofluorescence, myotubes were fixed in 100% methanol cooled to −20C for 5 min, after which they were blocked for 30 min with Odyssey block buffer (LI-COR). After blocking, we incubated cells with 1:250 dilution of rabbit anti-CD13 antibody (Abcam) overnight at 4C. After rinsing myotubes 3x, we incubated cells 1 h with 1:1000 secondary antibodies, donkey anti-rabbit 649 (DyLight). We captured fluorescence images using the same system as above (excitation: 655 nm, emission 670 nm). Fluorescence was measured with ImageJ (NIH).

### Proteomics

Mass spectrometric analysis was performed at the University of Kentucky, Proteomics Core Facility. This core facility is supported in part by funds from the Office of the Vice President for Research. Cellular locations of identified proteins were determined by database searches in Protein (NCBI) and UniProt (UniProt Consortium).

### Statistics

Prism 5.0b served as our statistics software (GraphPad Software). We used ANOVA, or repeated measures ANOVA, with Bonferroni post-test for multiple comparisons, and *t*-test for all other comparisons. Data are presented as mean ± SE. Statistical significance was defined as *p* < 0.05.

## Results

### LCM treatment induces loss of myotube width

**Figure 1:**
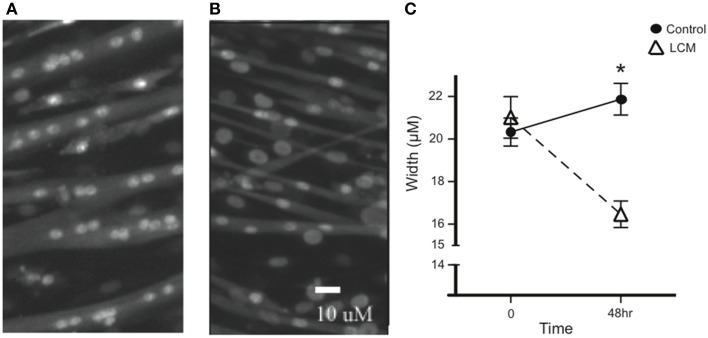
**LCM-induced loss of myotube width. (A)** Image of untreated myotubes. **(B)** Image of myotubes treated with LCM for 48 h. **(C)** Graph of width changes in control and LCM-treated myotubes (mean ± SE, *n* = 6, repeated measures ANOVA, ^*^P < 0.05).

To confirm the efficacy of our *in vitro* muscle-wasting model, we treated C2C12 myotubes with LCM and measured myotube width. We measured width before treatment and found that there was no difference in average width between groups. After 48 h of LCM treatment, we found that the average width in control myotubes increased, while the average width in LCM-treated myotubes decreased (Figures 1A–C).

### LCM treatment decreases total protein and myosin protein content

**Figure 2:**
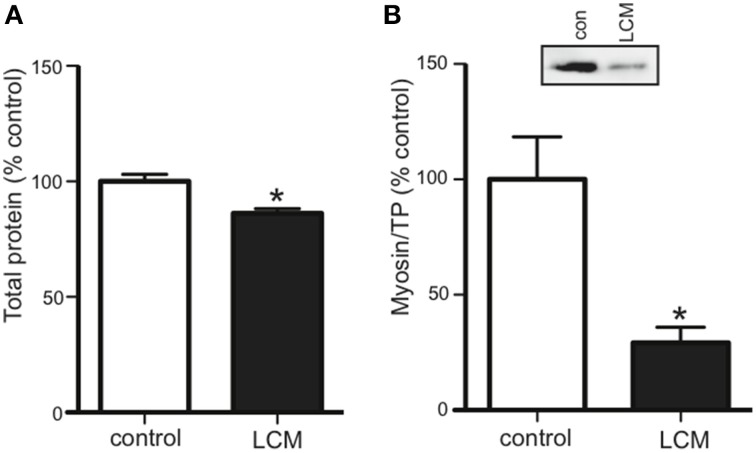
**LCM treatment decreases total protein and myosin in C2C12 myotubes**. 48 h of LCM treatment decreased **(A)** total protein by 14%, and **(B)** myosin content by 70% (mean ± SE *n* = 6, ^*^P < 0.05, *t*-test).

Cancer cachexia is known to preferentially degrade the myofibrillar protein, myosin (Acharyya et al., 2004; Banduseela et al., 2007; Ochala and Larsson, 2008). To confirm this outcome in our model, we treated myotubes for 48 h with LCM and measured total protein and myosin content via western blot. Compared to control, LCM treatment caused a loss of total protein and myosin (Figures 2A,B).

### Proteins in lewis lung cancer conditioned media

**Figure 3:**
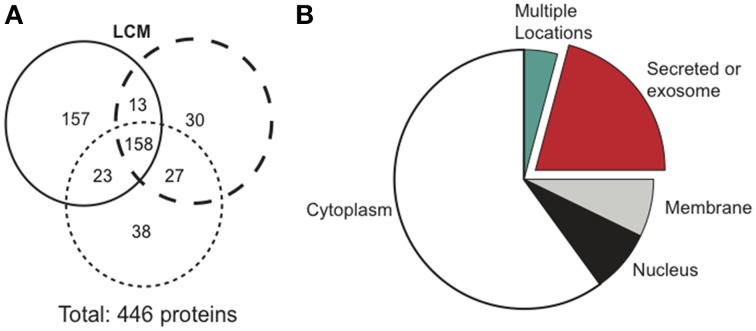
**Mass spectrometry results comparing protein distribution in LCM.(A)** Each circle represents one biological replicate of LCM and compares the number of proteins that the replicates shared in common. **(B)** Cellular location of the 158 proteins confirmed in LCM.

Cancerous tumors secrete factors that alter their surrounding environment. To investigate which tumor-derived proteins may contribute to cancer cachexia, we used mass spectrometry to examine three biological replicates of serum-free LCM. Mass spectrometry identified 446 total proteins, with 158 confirmed in all replicates (Figure 3A). We identified the cellular location of the 158 confirmed proteins and found that 33 were secreted (Figure 3B).

### Extracellular 14-3-3 proteins in LCM and their effect on myosin content

**Figure 4:**
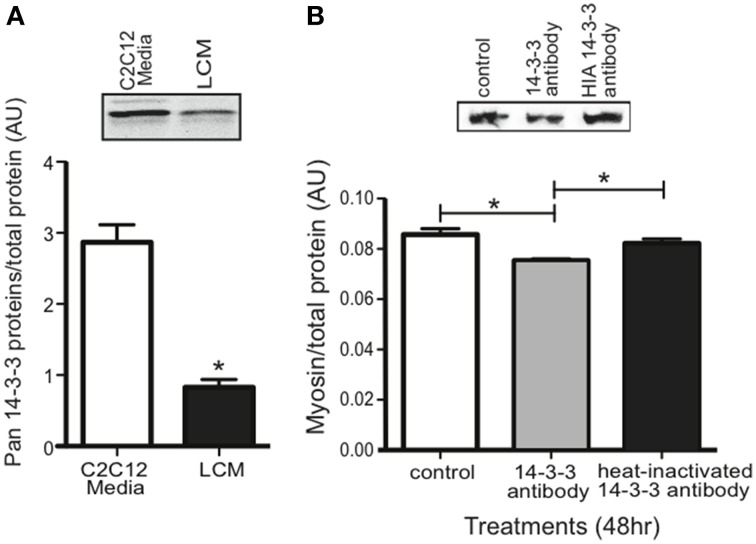
**14-3-3 content and its effect on myosin. (A)** Pan 14-3-3 content in C2C12-CM vs. LCM (mean ± SE *n* = 4, ^*^P < 0.05, *t*-test). **(B)** Myosin content decreased when extracellular 14-3-3 proteins were neutralized in myotube cell culture medium using an anti-pan14-3-3 antibody (mean ± SE, *n* = 4, ^*^P < 0.05, ANOVA with Bonferroni post test).

Our proteomics data showed that LCM contains all seven isoforms of 14-3-3 proteins, but any effect of extracellular 14-3-3 proteins on skeletal muscle was unknown. Using an anti-pan14-3-3 antibody, we assessed 14-3-3 content in media conditioned with untreated C2C12 myotubes (C2C12-CM) and in LCM via western blot. Compared to C2C12-CM, LCM contained less 14-3-3 content (Figure 4A). To assess whether extracellular 14-3-3 proteins affect myosin content in skeletal muscle, we neutralized 14-3-3 proteins in myotube culture medium using an anti-pan14-3-3 antibody. After 24 h, we examined myosin content via western blot. Neutralizing extracellular 14-3-3 proteins decreased myosin content compared to control myotubes, and myotubes treated with heat-inactivated anti-pan14-3-3 antibody (Figure 4B).

### CD13 receptor on C2C12 myotubes and adult skeletal muscle

**Figure 5:**
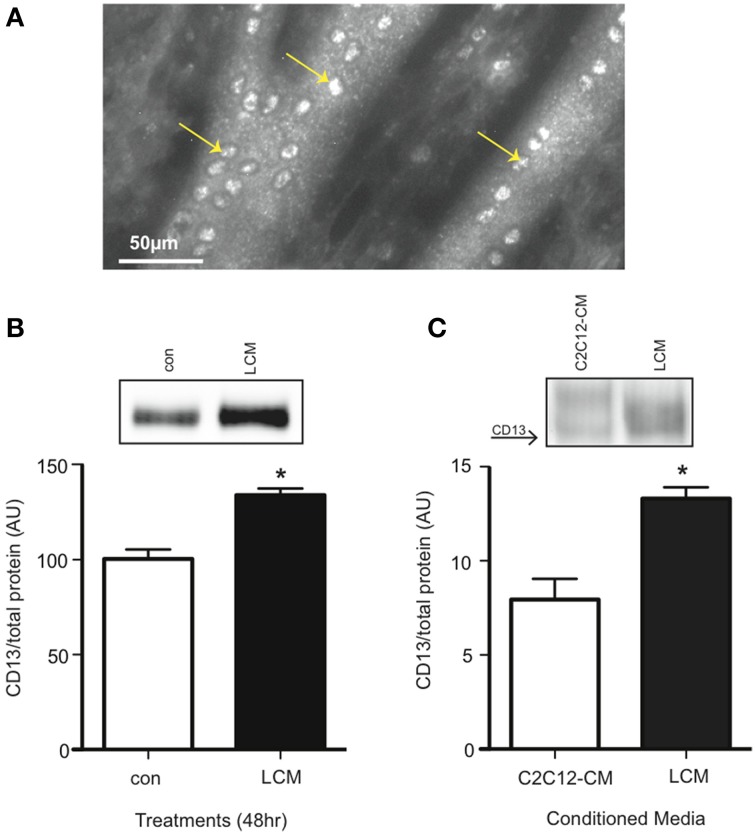
**CD13 content is elevated in LCM-treated C2C12 myotubes and LCM. (A)** Image of anti-CD13 antibody using a fluorescence microscope. **(B)** CD13 content in control vs. LCM-treated myotubes via western blot (mean ± SE, *n* = 6, ^*^P < 0.05, *t*-test). **(C)** CD13 content in C2C12-CM vs. LCM (mean ± SE, *n* = 4, ^*^P < 0.05, *t*-test).

CD13 is the proposed receptor for some extracellular isoforms of 14-3-3 (Ghaffari et al., 2010). CD13 is found on stem cells, including myoblasts, but its presence on differentiated skeletal muscle was not known (Ghosh et al., 2014; Rahman et al., 2014a,b). Using an anti-CD13 antibody, we examined non-permeabilized C2C12 myotubes for CD13 with a fluorescence microscope, and found it present (Figure 5A). We also confirmed CD13 content in myotubes via western blot. We found that LCM-treated myotubes had higher CD13 content compared to control (Figure 5B). Some pathologies show an increase in a soluble form of CD13 in plasma (Van Hensbergen et al., 2002; Piedfer et al., 2011; Wickstrom et al., 2011). Therefore, we tested CD13 content in C2C12-CM vs. LCM via western blot and found that LCM contained higher CD13 content (Figure 5C).

### Aminopeptidase inhibitor decreases myosin content in C2C12 myotubes

**Figure 6:**
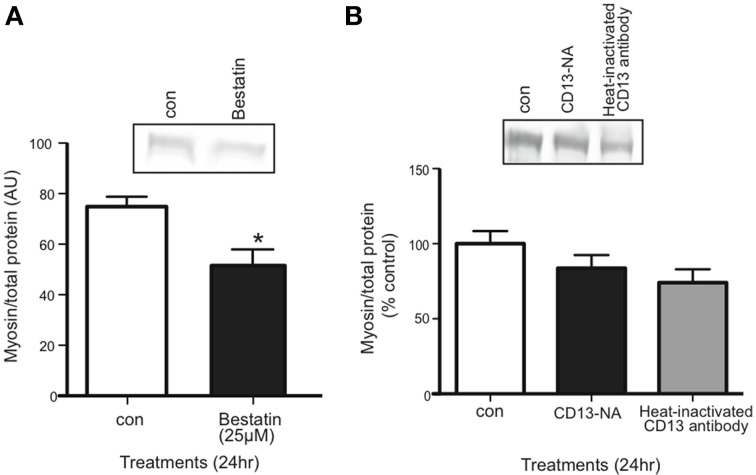
**Inhibition of CD13 with Bestatin, but not antibody neutralization, decreased myosin content in myotubes. (A)** Western blot comparing myosin content in control vs. Bestatin-treated myotubes (mean ± SE, *n* = 7, ^*^P < 0.05, *t*-test). **(B)** Western blot comparing myosin content in control myotubes vs. myotubes for which CD13 was neutralized in myotube culture medium or heat-inactivated CD23 antibody control (mean ± SE, *n* = 3, n.s., ANOVA).

Inhibiting the proposed receptor for extracellular 14-3-3 proteins should have the same affect on myotubes as neutralizing 14-3-3 proteins. We treated myotubes with Bestatin, an inhibitor that targets CD13 as well as aminopeptidase B and leukotriene A4 hydrolase. Using western blot, we found that 25 μM Bestatin decreased myosin content (Figure 6A). We also used an anti-CD13 antibody in myotube culture medium to neutralize CD13, but it did not affect myosin content (Figure 6B).

## Discussion

Cachexia presents in 50% of all cancer patients and contributes to 30% of all cancer deaths (Fox and Wang, 2007; Fearon, 2008; Tisdale, 2009). Efforts to identify the extracellular factors that initiate cachexia are ongoing. The current consensus is that cachexia syndrome is caused by multiple factors (Fearon et al., 2011; Tsoli and Robertson, 2013). Several well-established cachectic cytokines are elevated in the sera of cancer patients, including TNF-α, IL-6, and myostatin (Barton, 2001; Jespersen et al., 2006; Kayacan et al., 2006; White et al., 2013; Lokireddy et al., 2015). However, clinical trials for single-target inhibitors fail to fully alleviate muscle wasting (Mantovani et al., 2013; Tsoli and Robertson, 2013). These results suggest that unidentified factors may also be involved. Our goal was to identify new candidates that may contribute to muscle wasting. Our novel findings also show that: extracellular 14-3-3 proteins have a role in muscle maintenance; CD13 is present on differentiated skeletal muscle; and CD13 may act as the receptor for 14-3-3 proteins.

We began by validating our model using myotube size as a morphological endpoint, and myosin as the molecular endpoint. Myosin was chosen because it is preferentially degraded in cachexia, and as the major contractile protein in skeletal muscle, its loss can depress respiratory function, diminish strength, and increase fatigue (Acharyya et al., 2004; Du et al., 2004; Li et al., 2005; Tisdale, 2005; Zhang et al., 2011; Johns et al., 2013; Chacon-Cabrera et al., 2014). We confirmed that LCM treatment reduced size, total protein, and myosin content in myotubes (Figures 1, 2); (Acharyya et al., 2004; Du et al., 2004; Banduseela et al., 2007; Ochala and Larsson, 2008; Yano et al., 2008; Zhang et al., 2011; Goncalves et al., 2013; Stacchiotti et al., 2014). After establishing our model, we began identifying proteins in LCM.

Using mass spectrometry, we confirmed 158 proteins, including 33 secreted proteins (Figures 3A,B). Some proteins identified derived from intracellular compartments, a common occurrence in proteomic studies using conditioned media (Huang et al., 2006; Kim et al., 2008; Sardana et al., 2008; Planque et al., 2009; Yousefi et al., 2012). Due to high sensitivity of mass spectrometry, contents from the few cells that die during cell culture are identified. Alternatively, some intracellular proteins may be secreted via exosomes and are identified in conditioned media (Gross and Boutros, 2013; Raposo and Stoorvogel, 2013; Shimoda and Khokha, 2013; Aoi and Sakuma, 2014). Of the 33 secreted proteins, we identified 14-3-3 proteins as a promising cachexia-inducing candidate because they are involved in multiple signaling pathways and have over 200 known binding partners.

14-3-3 proteins are multi-functional and highly conserved binding proteins (Freeman and Morrison, 2011; Gardino and Yaffe, 2011; Kleppe et al., 2011; Obsil, 2011). They include seven isoforms, which are routinely found in the intracellular environment affecting diverse signaling pathways such as metabolism, growth, and apoptosis (Freeman and Morrison, 2011; Gardino and Yaffe, 2011; Kleppe et al., 2011; Obsil, 2011). However, several recent studies have shown that some isoforms act in an extracellular manner to activate signaling cascades. Maksymowych et al. reported that extracellular 14-3-3η was elevated in patients with rheumatoid arthritis (RA). THP-1 cells treated with 14-3-3η showed increased phosphorylation of ERK1/2 and JNK/SAPK and increased production of inflammatory transcripts, IL-1β, IL-6, and MMP-1 (Maksymowych et al., 2014). In another study, Ghaffari et al. found that extracellular 14-3-3σ increased MMP-1 content in fibroblasts (Ghaffari et al., 2010). Similarly, Asdaghi et al. found that extracellular 14-3-3α/β induced MMP-1 transcript in lung fibroblasts (Asdaghi et al., 2012). Notably, they also found that media conditioned with lung cancer cell line, A549, and its control, bronchial epithelial cell line HS24, both contained 14-3-3α/β; however, the results for each cell line were presented separately, making it impossible to compare extracellular 14-3-3 content.

In our study, we compared 14-3-3 content between LCM and C2C12 myotube conditioned media. When we confirmed the presence of 14-3-3 proteins in LCM via western blot, we found that LCM contained lower pan14-3-3 content than C2C12-CM (Figure 4A). In order to determine whether depletion of extracellular 14-3-3 content might contribute to cachexia, we treated myotube culture media with an anti-pan14-3-3 antibody. Neutralization of extracellular 14-3-3 proteins decreased myosin content in myotubes (Figure 4B). These data suggest that extracellular 14-3-3 proteins are necessary for maintaining myosin content in skeletal muscle, however a receptor for extracellular 14-3-3 proteins in skeletal muscle was unknown.

Previous work by Ghaffari et al. in fibroblasts showed that CD13 may act as the receptor for some isoforms of 14-3-3. In their 2010 study, the authors used immunofluorescence microscopy and found that 14-3-3σ co-localized with CD13 (Ghaffari et al., 2010). CD13 is found on many types of stem cells, including skeletal muscle satellite cells (Rahman et al., 2014a,b). Using an animal model of ischemic hind-limb injury, Rahman et al. found that CD13^KO^ had impaired muscle regeneration compared to WT control mice (Rahman et al., 2014a). These findings showed that CD13 is present on muscle satellite cells and that it affects skeletal muscle, but its presence on differentiated skeletal muscle was unknown. Using immunofluorescence microscopy, we confirmed the presence of CD13 on differentiated skeletal muscle, which is in itself a novel finding (Figure 5A). Our results also showed that LCM treatment increased CD13 content in myotubes (Figure 5B). Van Hensbergen et al. found elevated soluble CD13 (sCD13) in the plasma of cancer patients, and suggested that tumors shed CD13 from the plasma membrane (Van Hensbergen et al., 2002). When we examined LCM vs. C2C12-CM for sCD13, we found that LCM contained elevated sCD13 content (Figure 5C). These novel data confirm the presence of CD13 on differentiated skeletal muscle and in conditioned media from both lung tumors and skeletal muscle. We speculate that sCD13 may bind 14-3-3 proteins in the same manner that soluble TNFα receptors capture TNFα: soluble CD13 may be the mechanism for extracellular 14-3-3 depletion.

With the presence of CD13 confirmed in differentiated myotubes, we treated myotubes with Bestatin, a potent aminopeptidase inhibitor, to determine if CD13 is the receptor for 14-3-3 proteins in skeletal muscle. In theory, CD13 inhibition should affect skeletal muscle in the same manner as depletion of 14-3-3 proteins. In fact, 25 μM Bestatin did decrease myosin content, which to our knowledge is a novel finding (Figure 6A). These results show that aminopeptidases are involved in skeletal muscle maintenance, but as Bestatin has multiple targets, we needed to inhibit CD13 specifically. However, CD13 neutralization using an anti-CD13 antibody did not affect myosin content (Figure 6B). Interestingly, Ghaffari et al. had the opposite result: when they used Bestatin to inhibit the effects of extracellular 14-3-3σ via CD13, it had no effect (Ghaffari et al., 2010). Their results may indicate that CD13 is not the receptor for 14-3-3σ despite co-localization, or, as the authors suggested, that the site of CD13 enzymatic action regarding MMP-1 may be at an alternate site from that which Bestatin targets. There are several epitopes on CD13 targeted by different monoclonal antibodies (Xu et al., 1997; Mina-Osorio, 2008; Piedfer et al., 2011). It is possible that the antibody we used labeled CD13 without affecting the site where 14-3-3 proteins bind.

## Conclusions

Extracellular 14-3-3 proteins appear to have a role in maintaining skeletal muscle mass, and some isoforms may act as previously unidentified myokines. CD13 is a candidate receptor for 14-3-3 proteins, and its soluble form may be the mechanism for 14-3-3 diminishment in LCM. Future studies should include determining which individual isoforms of 14-3-3 act in the extracellular space to affect skeletal muscle mass, and what role soluble CD13 plays in differentiated skeletal muscle. This study proposes a role for extracellular 14-3-3 proteins in skeletal muscle and is an initiating point for future studies delineating this previous unidentified pathway.

## Author contributions

JBM, JSM, and EH performed experiments and analyzed data; JBM prepared figures, JBM drafted manuscript, JBM, JSM and FA edited and revised manuscript; JBM, JSM, and FA conceived experimental design; JBM, JSM, and FA approved final draft.

### Conflict of interest statement

The authors declare that the research was conducted in the absence of any commercial or financial relationships that could be construed as a potential conflict of interest.

## Abbreviations

LCM: Lewis lung carcinoma conditioned medium
PAGE: polyacrylamide gel electrophoresis.
